# The efficacy and immune-mediated safety of PD-1/PD-L1 combined with neoadjuvant chemotherapy in triple-negative breast cancer: a meta-analysis

**DOI:** 10.3389/fonc.2025.1635418

**Published:** 2025-08-07

**Authors:** Xiao Yan, Qi Lv, Jiangzhuo Wu, Jiang Fang, Lin Peng, Xiaobo Zhao

**Affiliations:** ^1^ Department of Thyroid and Breast Surgery/School of Clinical Medicine, North Sichuan Medical College/Affiliated Hospital of North Sichuan Medical College, Nanchong, Sichuan, China; ^2^ Department of Surgical Center, Affiliated Hospital of North Sichuan Medical College, Nanchong, Sichuan, China

**Keywords:** PD-1/PD-L1 inhibitors, neoadjuvant chemotherapy; triple-negative breast cancer, immune checkpoint inhibitors, meta-analysis, systematic review

## Abstract

**Background:**

The efficacy and immune-mediated safety of PD-1/PD-L1 inhibitors in triple-negative breast cancer (TNBC) remain controversial. Given TNBC’s aggressive biology and poor prognosis, definitive evidence is urgently needed. We performed this meta-analysis to comprehensively assess the benefits and safety of these inhibitors by examining clinical trial data for TNBC.

**Methods:**

Up until October 25, 2024, a thorough search was done in the PubMed, Embase, and Cochrane databases to find research assessing PD-1/PD-L1 inhibitors in treating TNBC. This study ultimately included 8 randomized controlled trials involving 5,512 patients. Pathological complete response (pCR), progression-free survival (PFS), overall survival (OS), event-free survival (EFS), and immune-related adverse events (irAEs) were among the primary objectives, which defined as adverse drug reactions affecting various organ systems due to immune system activation, were graded according to CTCAE v5.0 criteria.

**Results:**

The combination of PD-1/PD-L1 inhibitors with neoadjuvant chemotherapy significantly increased pCR rates by 77% compared to chemotherapy alone (OR=1.77, 95% CI: 1.28-2.45, P<0.01). Subgroup analyses indicated that the benefit of pCR was more evident in patients with lymph node positivity(OR=2.57,95% CI:1.76–3.75, P < 0.01). For EFS, the integration of immune checkpoint inhibitors(ICIs) combination therapy decreased the possibility of events by 35% (HR=0.65,95%CI:0.54–0.80, P< 0.01), with notable benefits observed in earlier-stage (T1-T2) patients(HR= 0.53, 95%CI:0.40–0.70, P < 0.01). Similarly, PFS was improved in the experimental group for both ITT (HR=0.79,95% CI, 0.71–0.88, P<0.01) and PD-L1 positive populations (HR=0.71,95%CI:0.63–0.81, P < 0.01). However, the incidence of irAEs was significantly higher in the ICIs group compared to the neoadjuvant chemotherapy group (OR=2.77,95% CI:1.93–3.96, P < 0.01).

**Conclusion:**

With lymph node status acting as a crucial predictor, the combination of PD-1/PD-L1 inhibitors and neoadjuvant chemotherapy dramatically improves pCR and EFS in TNBC. Additionally, it improves OS and PFS, although at the cost of an increased incidence of irAEs. These findings offer insightful information for upcoming clinical trial designs, economic evaluations, and clinical decision-making in TNBC treatment.

**Systematic review registration:**

https://www.crd.york.ac.uk/prospero/, identifier CRD42025640551.

## 1 Introduction

Breast cancer, which stands as the most widespread malignancy affecting women globally, comprises roughly 30% of all cancers diagnosed in women, carrying a mortality-to-incidence ratio of 15%. With an occurrence frequency of 27 cases for every 100,000 people in East Asia and Africa, the condition persists as a significant area of interest in medical research, specifically in relation to treatment approaches and disease outcomes (1). Luminal A, luminal B, HER-2 overexpressing, and TNBC subtypes are the main classifications for breast cancer based on the expression of particular biomarkers like Ki67, HER-2, progesterone receptor (PR), estrogen receptor (ER) (2). Among these subtypes, Luminal A and Luminal B collectively account for approximately 70% of cases, while HER-2 enriched and TNBC represent 15% and 15 to 20% of cases, respectively (3).

Among the numerous subtypes of cancer, TNBC stands out as particularly distinct. Defined by the absence of expression of the ER, PR, and HER-2, TNBC accounts for 15% to 20% of all breast cancers (4), which exhibits high heterogeneity and complex biological characteristics, posing challenges in establishing a unified biological model. Characterized by high proliferative activity, TNBC is known for its aggressive behavior, with earlier relapses and poorer survival outcomes (5, 6). While neoadjuvant chemotherapy remains the primary treatment for TNBC, recent molecular analyses have identified potential therapeutic targets, examples including immune checkpoint inhibitors, offering novel treatment strategies for this challenging subtype (7).

**Figure 1:**
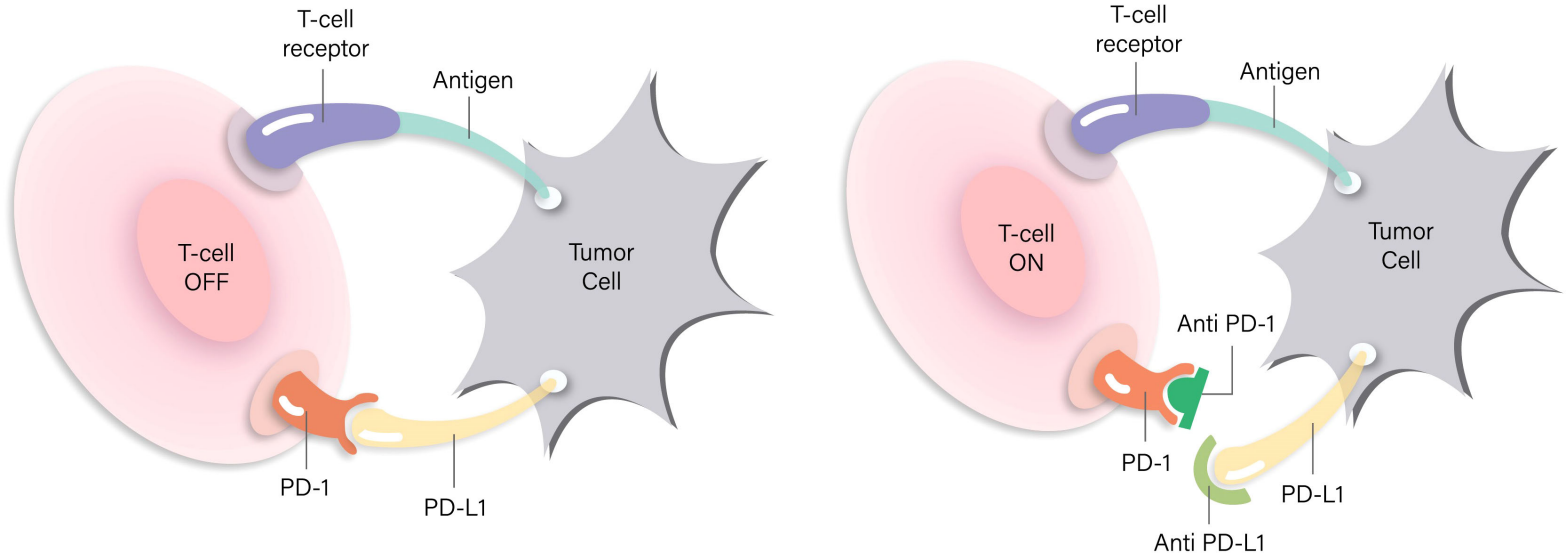
Schematic diagram of the mechanism of PD-1/PD-L1 inhibitors.

For TNBC immunotherapy, agents that target the PD-1/PD-L1 axis have been a major area of research interest. PD-1, alternatively referred to as Programmed Death-1, represents an immunosuppressive receptor that is mostly seen on the surface of T cells that have been stimulated. When it receives signals from its corresponding ligand, PD-L1, it triggers T-cell exhaustion and acts as a brake to prevent overstimulation of the immune system (8). It binds to its recognized ligand, PD-L1. Through its interaction with PD-L1 located on the surfaces of tumors and immune cells, PD-1 signaling suppresses T-cell activation during the immune response’s effector phase. This ultimately hinders the proliferation and activation of T cells while augmenting antigen presentation by tumor cells, thereby facilitating the recognition and targeting of these tumor cells by immune cells (**Figure 1**). In TNBC, PD-L1 is observed to have relatively high expression levels affecting immune cells as well as malignant cells, and its abundance is closely associated with the prognosis of PD-1 inhibitor therapy (9). Therefore, immunotherapy targeting the PD-1/PD-L1 pathway presents a promising new therapeutic approach for TNBC. The monoclonal antibody targeting PD-1, pembrolizumab, has demonstrated encouraging antitumor activity along with a tolerable safety profile when administered as a monotherapy in various tumor types, specifically including metastatic triple-negative breast cancer (10, 11).

Numerous PD-1/PD-L1 inhibitors, including atezolizumab and pembrolizumab, have demonstrated promising clinical activity. Despite advancements from clinical studies evaluating PD-1/PD-L1 inhibitors in TNBC, the accumulated evidence-based data remains limited compared to other cancers, and the results are often controversial. Concerns persist regarding their efficacy and immune-related safety. PD-1/PD-L1 inhibitors can trigger various immune-related adverse events. Pneumonitis occurs in approximately 3% of patients, with clinical studies reporting three fatal cases. Dermatologic toxicities are among the most common side effects, including rash in about 12% of cases and vitiligo in 3%. Gastrointestinal complications primarily manifest as diarrhea, affecting roughly 11% of treated individuals. Additionally, endocrine disorders such as thyroid dysfunction develop in approximately 3% of patients receiving this immunotherapy (12). Additionally, the effectiveness and immune-associated safety of combining PD-1/PD-L1 inhibitors with neoadjuvant chemotherapy for TNBC remains uncertain. Hence, the objective of this study is to conduct a meta-analysis to comprehensively assess the effectiveness and immune-associated safety profile of PD-1/PD-L1 inhibitors when combined with neoadjuvant chemotherapy for the treatment of TNBC. This approach aims to provide accurate direction for clinical diagnosis and therapeutic interventions.

This meta-analysis systematically evaluates the efficacy and safety of PD-1/PD-L1 inhibitors in TNBC by synthesizing evidence across key endpoints including pCR, EFS, OS, PFS, and irAEs. By quantifying treatment benefits and risks across clinically relevant outcomes, this study aims to provide evidence-based guidance for immunotherapy integration in TNBC management.

## 2 Methods

### 2.1 Data sources and search strategy

The present investigation was carried out in compliance with the PRISMA (Preferred Reporting Items for Systematic Reviews and Meta-Analyses) framework criteria, as outlined by Moher, and was registered in PROSPERO (ID: CRD42025640551). A thorough literature search was conducted separately by two researchers in the Cochrane Library, PubMed, and Embase. The search was tailored using the following keywords: “Programmed Cell Death 1 Receptor,” “Immune Checkpoint Inhibitors,” “B7-H1 Antigen,” “Neoadjuvant Therapy,” and “Breast Neoplasms.” The search period concluded on October 25th, 2024, and was restricted to English-language publications.

### 2.2 Eligibility criteria and study selection

The inclusion criteria were rigorously defined to encompass original controlled trials with efficacy outcomes, including OS, PFS, pCR, EFS, and irAEs, with the requirement that studies report at least one prespecified outcome for inclusion, but not necessarily all key outcomes. Participants included were adults diagnosed with TNBC who received PD-1/PD-L1 inhibitors either as a monotherapy or in combination with neoadjuvant chemotherapeutic agents. Conference data were also included based on the following criteria: (1) presented at major oncology conferences (ESMO, ASCO, SABCS); (2) provided complete methodological details; and (3) contained sufficient outcome data for meta-analysis. The inclusion criteria for this study were as follows: randomized controlled clinical trials in Phase 2 or 3, and patients who had histologically and cytologically verified TNBC. The experimental group was given neoadjuvant chemotherapy together with PD-1/PD-L1 inhibitors, with the control group only receiving neoadjuvant chemotherapy alone. The following outlined the exclusion criteria: studies that were not randomized controlled trials (RCTs), including single-arm trials, retrospective analyses, observational retrospective studies, case reports, meta-analyses, and animal experiments; literature where extractable data was not available, whether directly or indirectly; and publications written in languages other than English.

### 2.3 Data extraction

For every trial included in the study, essential details were collected, such as the trial’s title, primary author, year of publication, total number of patients enrolled, along the clinical and pathological traits of the study participants. Specific data points included types of neoadjuvant chemotherapy and checkpoint inhibitors used, pCR data in the ITT population, and subgroup analyses based on age, PD-L1 expression, T stage, and regional lymph nodes. We adopted the following approaches to address variations in pCR and PD-L1 positivity definitions across studies:

(1) applying the most stringent pCR criteria (ypT0/Tis ypN0) when studies reported multiple definitions;(2) accounting for differences in PD-L1 testing methods by analyzing data according to their original trial protocols. Additionally, Hazard Ratios (HRs) and 95% Confidence Intervals (CIs) for EFS, PFS, and OS were extracted, along with subgroup analyses based on PD-L1 expression. Incidence rates of immune-mediated adverse events in the ITT population were also recorded. In addition, to ensure rigorous study selection, two independent reviewers performed both title/abstract screening and full-text review in parallel. When initial discrepancies arose between the reviewers regarding study eligibility or data extraction, we implemented a structured reconciliation process involving: (1) systematic re-evaluation of the original articles through joint discussion; and (2) if consensus remained unattainable, adjudication by a third senior reviewer with expertise in the field.

### 2.4 Bias assessment

**Figure 2:**
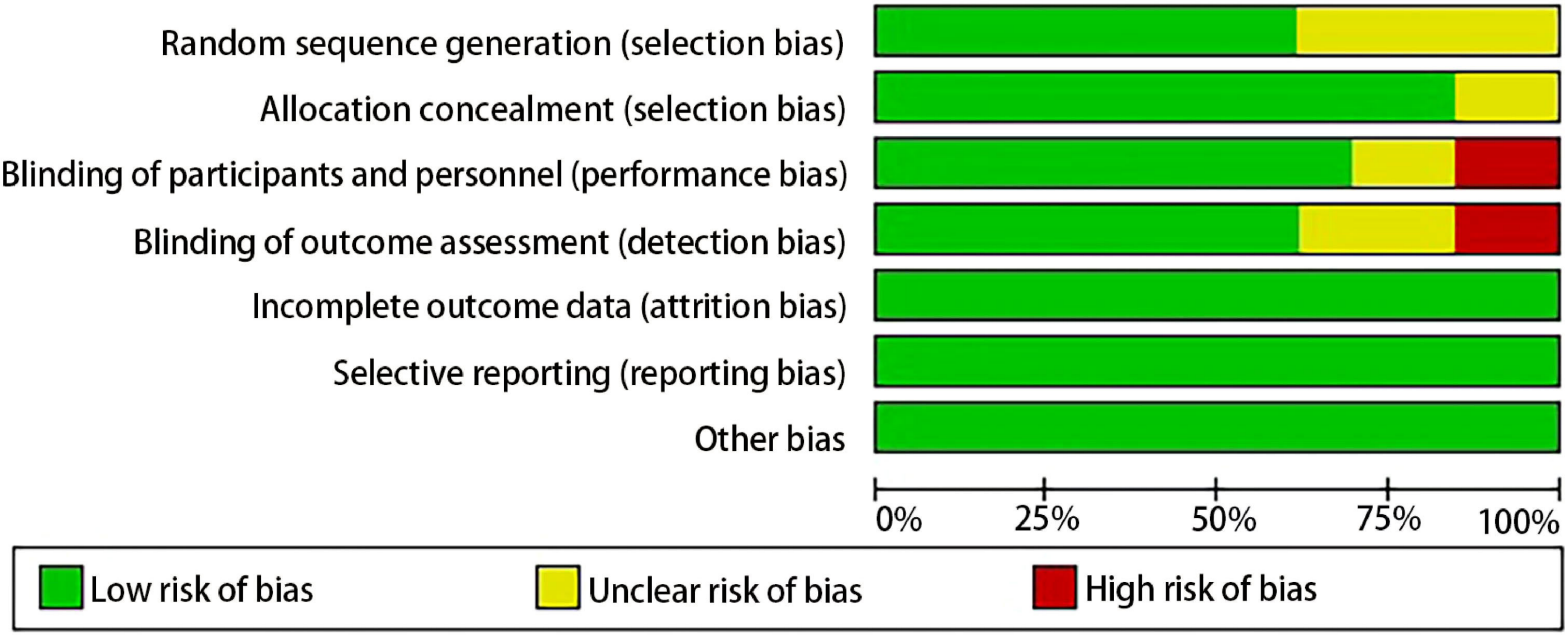
Risk of bias summary: review for the enrolled study.

Two researchers independently evaluated each included study’s risk of bias using the Cochrane Handbook’s recommendations. Blinding study participants and staff (performance bias), blinding during result evaluation (detection bias), creating random sequences (selection bias), hiding allocation (selection bias), providing incomplete information on results (attrition bias), biased reporting (reporting bias), and other biases were among the main sources of bias that were investigated. The Cochrane risk bias evaluation’s findings are displayed in **Figure 2**.

### 2.5 Statistical analysis

Statistical evaluations were conducted utilizing RevMan 5.4 software. The generic inverse variance method was employed for calculating and recording the HR and the corresponding standard error (SE) for PFS, OS, and EFS data. Dichotomous data types were selected for outcomes such as pCR and irAEs. The heterogeneity of the studies was estimated using the I² statistic. Random-effects models were applied if P < 0.1 or I² > 50%; otherwise, fixed-effects models were used. The threshold for statistical significance was established as P < 0.05.

## 3 Results

### 3.1 Search results and main characteristics of included trials

**Figure 3:**
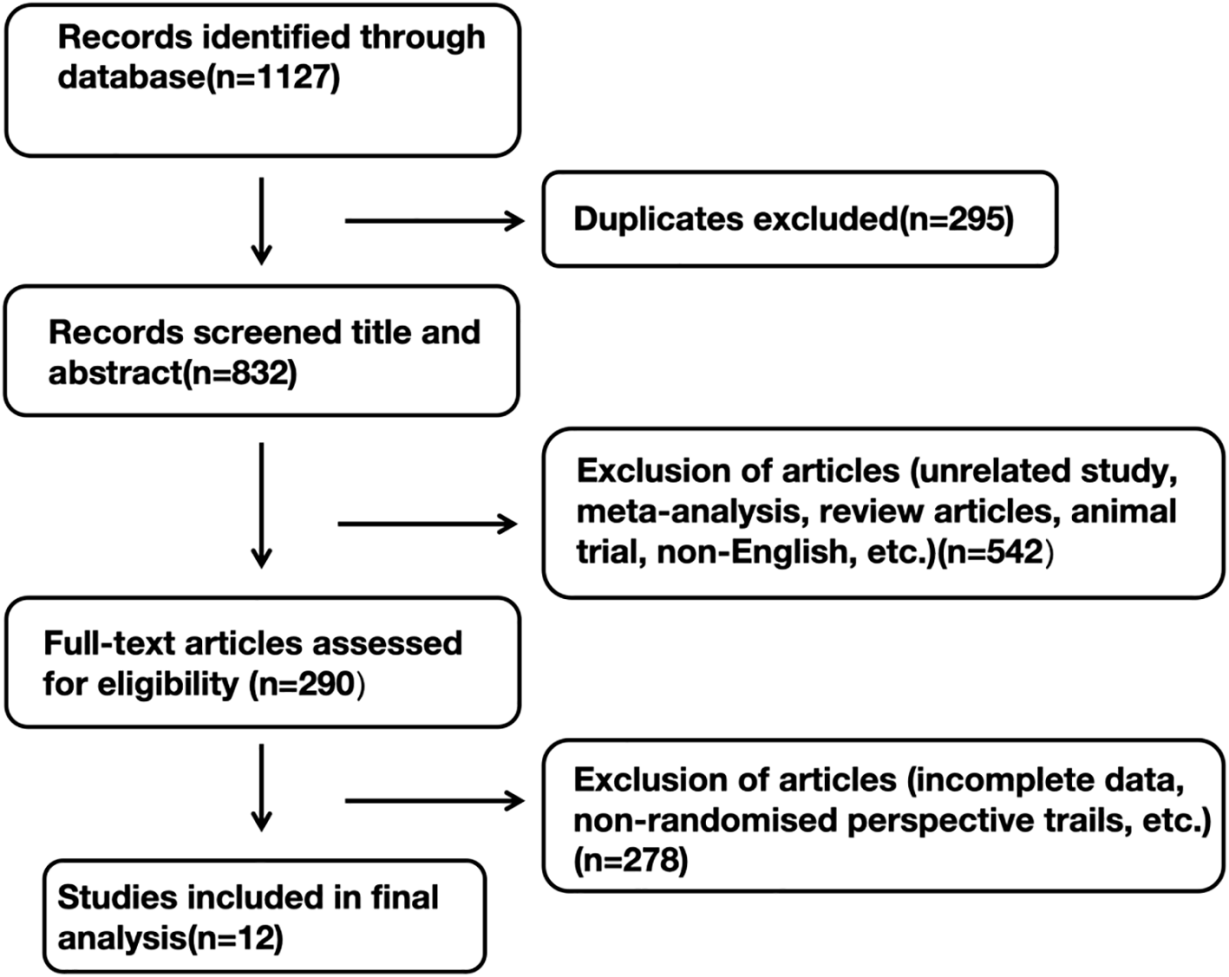
Flow diagram of study inclusion and exclusion.

Using the search approach, 1127 studies in total were found. Following abstract screening and full-text review to exclude duplicates and ineligible studies, eight RCTs involving 5,512 patients with TNBC met the inclusion criteria and were included in this meta-analysis [IMpassion031 (13, 14); KEYNOTE-355 (15); NeoTRIP (16); KEYNOTE-522 (17–19); GeparNuevo (20, 21); IMpassion131 (22); I-SPY2 (23); IMpassion130 (24)]. Notably, at the 2023 ESMO meeting, the EFS data from IMpassion031 were presented, whereas every other study was a complete, original publication. The literature finding and choosing process is depicted in **Figure 3**.

The studies included in this review were published during the period from 2018 to 2024. Among the eight RCTs, three investigated PD-1 inhibitors (I-SPY2;KEYNOTE-522;KEYNOTE-355), while five focused on PD-L1 inhibitors (IMpassion031; NeoTRIP; GeparNuevo; IMpassion131; I-SPY2). Four trials (NeoTRIP; IMpassion131; Keynote-355; IMpassion130) used a non-anthracycline-based regimen, while neoadjuvant chemotherapy (NACT) based on taxanes and anthracyclines, without or with platinum medicines, was employed in four investigations.

In terms of outcomes, five studies reported all-patient pCR, three provided lymph node status (N stage) specific pCR, and two included T stage-specific pCR. Additionally, three studies assessed pCR outcomes by PD-L1 status (positive/negative). As for efficacy endpoints, four studies documented ITT population EFS, three provided lymph node status-specific EFS, and three included EFS based on PD-L1 status. Two studies also reported EFS outcomes based on the T stage. Moreover, five studies reported OS outcomes in the ITT population, and four focused on PD-L1 positive subgroups. Three studies provided PFS data for both the ITT population and PD-L1 positive subgroups. Finally, four studies documented irAEs.

The treatment regimens varied across studies, and **Table 1** summarizes the main characteristics of the included trials.

**Table 1:** The main characteristics of the studies included in the meta-analysis.

Author	Year	Tiral	Study design	Chemotherapy agent	Sample size	Efficacy outcomes	PD-1/PD-L1
Elizabeth	2020	IMpassion031	double-blind, randomised	nab-paclitaxel, doxorubicin, cyclophosphamide	333	pcr	atezolizumab
Javier Cortes (15)	2020	KEYNOTE-355	randomised, double-blind	nab-paclitaxel;paclitaxel/ gemcitabine plus carboplatin	847	pfs	pembrolizumab
P. Schmid (19)	2020	KEYNOTE-522	randomized, double-blind trial	paclitaxel and carboplatin, doxorubicin–cyclophosphamide/epirubicin–cyclophosphamide	1174	pcr	pembrolizumab
L. Gianni (16)	2022	NeoTRIP	randomized, open-label study	carboplatin and nab-paclitaxel	280	pcr	atezolizumab
P. Schmid (18)	2022	KEYNOTE-522	randomized, double-blind trial	paclitaxel and carboplatin	1174	efs	pembrolizumab
P. Schmid (17)	2024	KEYNOTE-522	randomized, double-blind trial	paclitaxel and carboplatin	1174	os, efs	pembrolizumab
S. Loibl (21)	2022	GeparNuevo	randomized, double-blind trial	nab-paclitaxel	174	idfs, pcr	durvalumab
D. Miles (22)	2021	IMpassion131	randomized, double-blind trial	paclitaxel	651	pfs, os	atezolizumab
S. Loibl (20)	2019	GeparNuevo	randomized, double-blind trial	nab-paclitaxel,epirubicin /cyclophosphamide	174	pcr	durvalumab
C. Barrios	2023	IMpassion031	randomized, double-blind trial	nab-paclitaxel, doxorubicin, cyclophosphamide	333	efs,dfs,os	atezolizumab
Nanda (23)	2020	ispy2	adaptive randomized, open-label study	paclitaxel,doxorubicin	1151	pcr	pembrolizumab
P. Schmid (24)	2018	IMpassion130	randomized, double-blind trial	nab-paclitaxel	902	efs,os	atezolizumab

### 3.2 pCR and subgroup analyses for pCR

**Figure 4:**
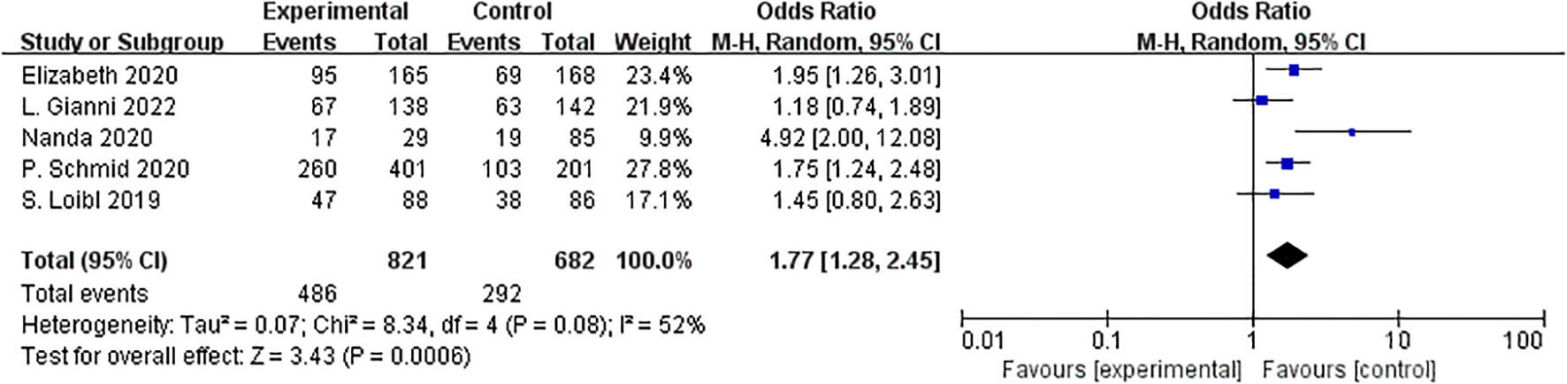
Forest plot of odds ratios for pCR.

Combination therapy with immune ICIs significantly raised the odds of pCR by 77% compared to neoadjuvant chemotherapy alone (OR= 1.77, 95%CI:1.28–2.45, P < 0.01, I^2^ = 52%) (**Figure 4**).

**Figure 5:**
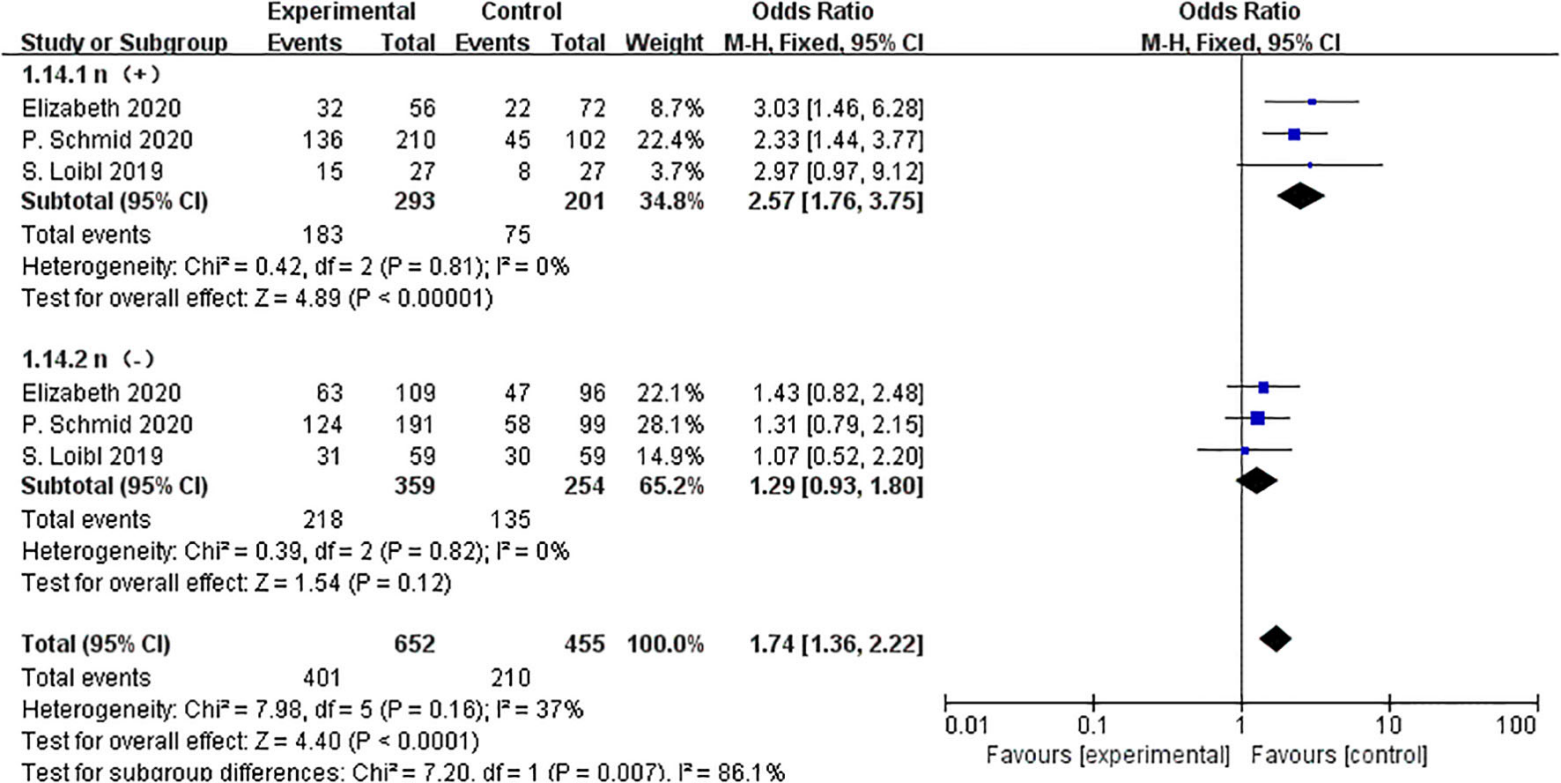
Forest plot of odds ratios comparing pCR in patients for N-stage subgroups.

Three trials with 1,107 participants provided data on pCR according to lymph node condition. Subgroup analysis revealed that NACT combined with ICIs greatly increased the rate of pCR in patients with positive lymph nodes (OR=2.57, 95%CI:1.76–3.75, P < 0.01, I^2^ = 0). However, no significant benefit was observed in patients with lymph node-negative (OR=1.29, 95% CI: 0.93–1.80, P =0.12, I^2^ = 0). There was a statistically significant correlation between pCR and lymph node status. (P < 0.01) (**Figure 5**). These findings suggest that lymph node-positive TNBC patients may derive substantial benefits from immune checkpoint inhibitors in achieving pathological complete response.

**Figure 6:**
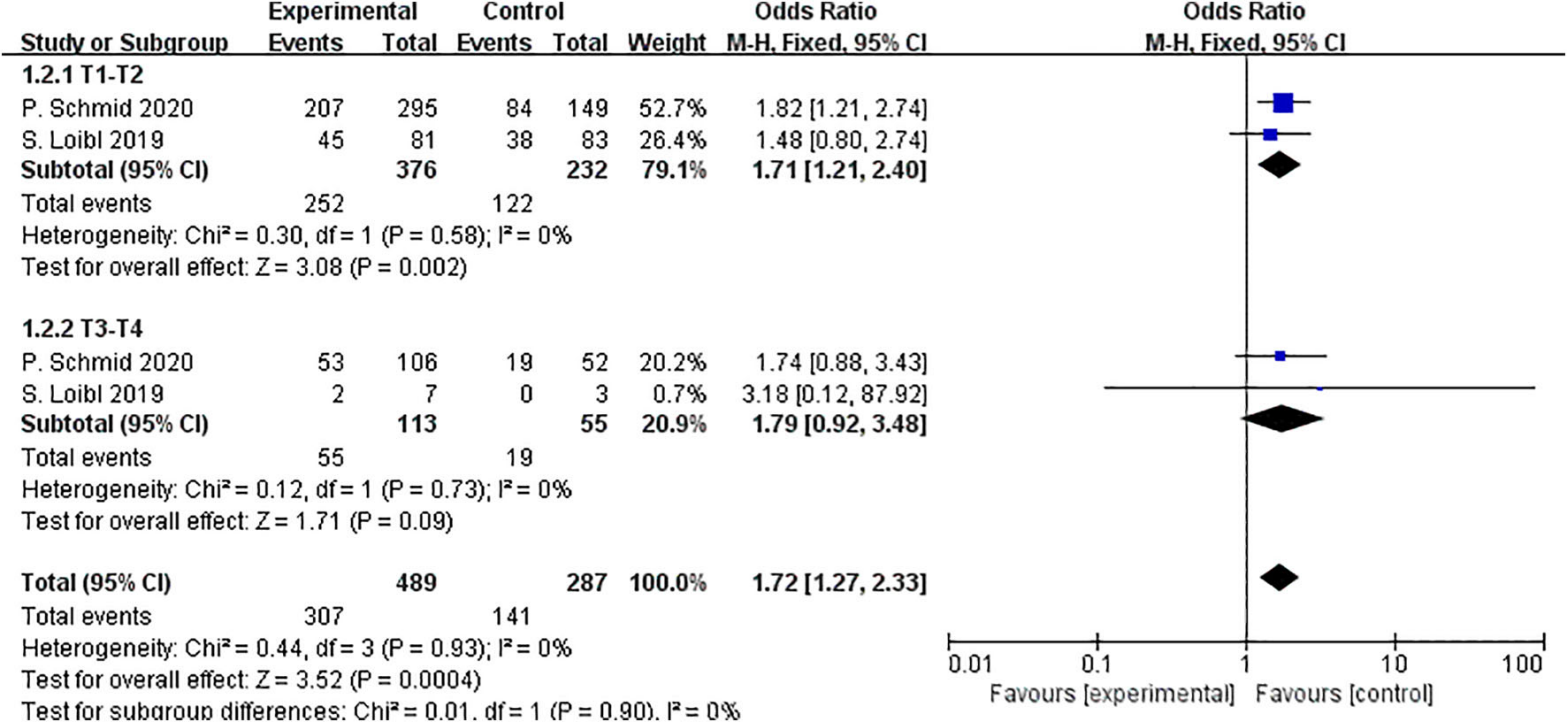
Forest plot of odds ratios comparing pCR in patients for T stage subgroups.

Two RCTs involving 776 patients provided subgroup analysis results based on the T stage of tumors. The pooled OR for patients with T1-T2 tumors was 1.71 (95% CI: 1.21–2.40, P < 0.01, I^2^ = 0), while the OR for patients with T3-T4 tumors was 1.79 (95% CI: 0.92–3.48, P =0.09, I^2^ = 0). The relationship between pCR and T stage was not significant (P = 0.90) (**Figure 6**).

**Figure 7:**
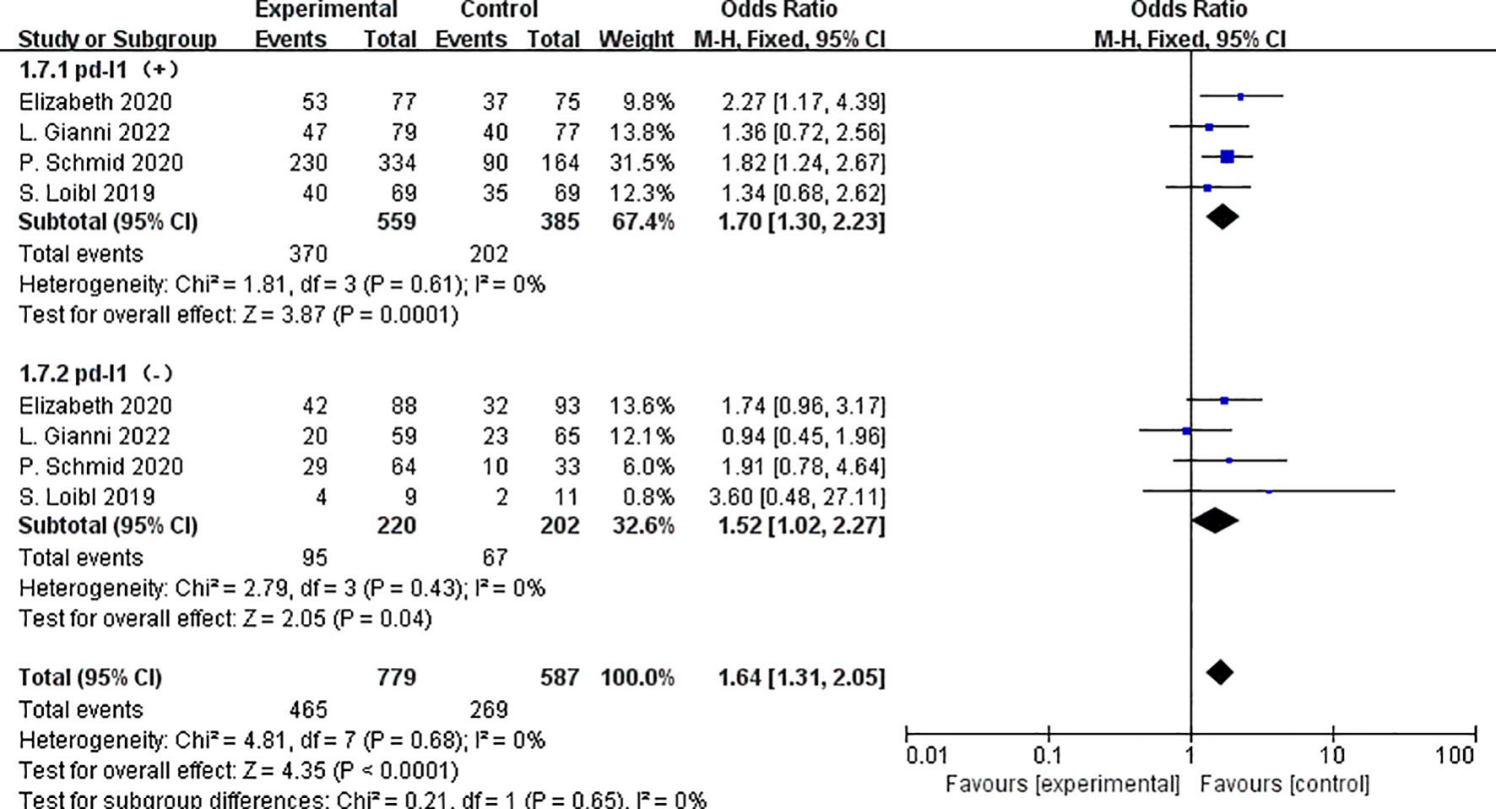
Forest plot of odds ratios for PD-L1 expression subgroup comparison of pCR in patients.

Based on PD-L1 expression, pCR outcomes were reported in four investigations involving 1,366 patients. PD-1/PD-L1 inhibitors were linked to an increased pCR rate, irrespective of PD-L1 expression status: the OR was 1.70 for patients with PD-L1 positive tumors (OR=1.70,95%CI:1.30–2.23, P<0.01, I2 = 0) and 1.52 for patients with PD-L1 negative populations(OR=1.52, 95%CI:1.02–2.27, P=0.04, I2 = 0). There was no statistically significant difference in the pCR rates between patients with PD-L1 positive and PD-L1 negative populations. (P = 0.65) (**Figure 7**).

### 3.3 EFS and subgroup analyses for EFS

**Figure 8:**
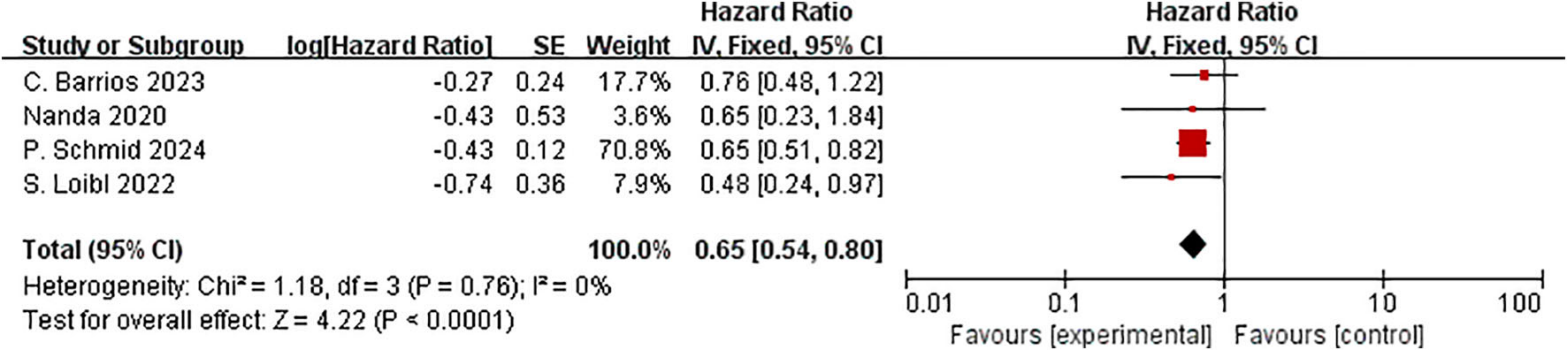
Forest plot of hazard ratios for EFS in patients.

Combination therapy with ICIs significantly reduced the chance of events by 35% compared to neoadjuvant chemotherapy alone (HR= 0.65, 95% CI:0.54–0.80, P < 0.01, I^2^ = 0) (**Figure 8**).

**Figure 9:**
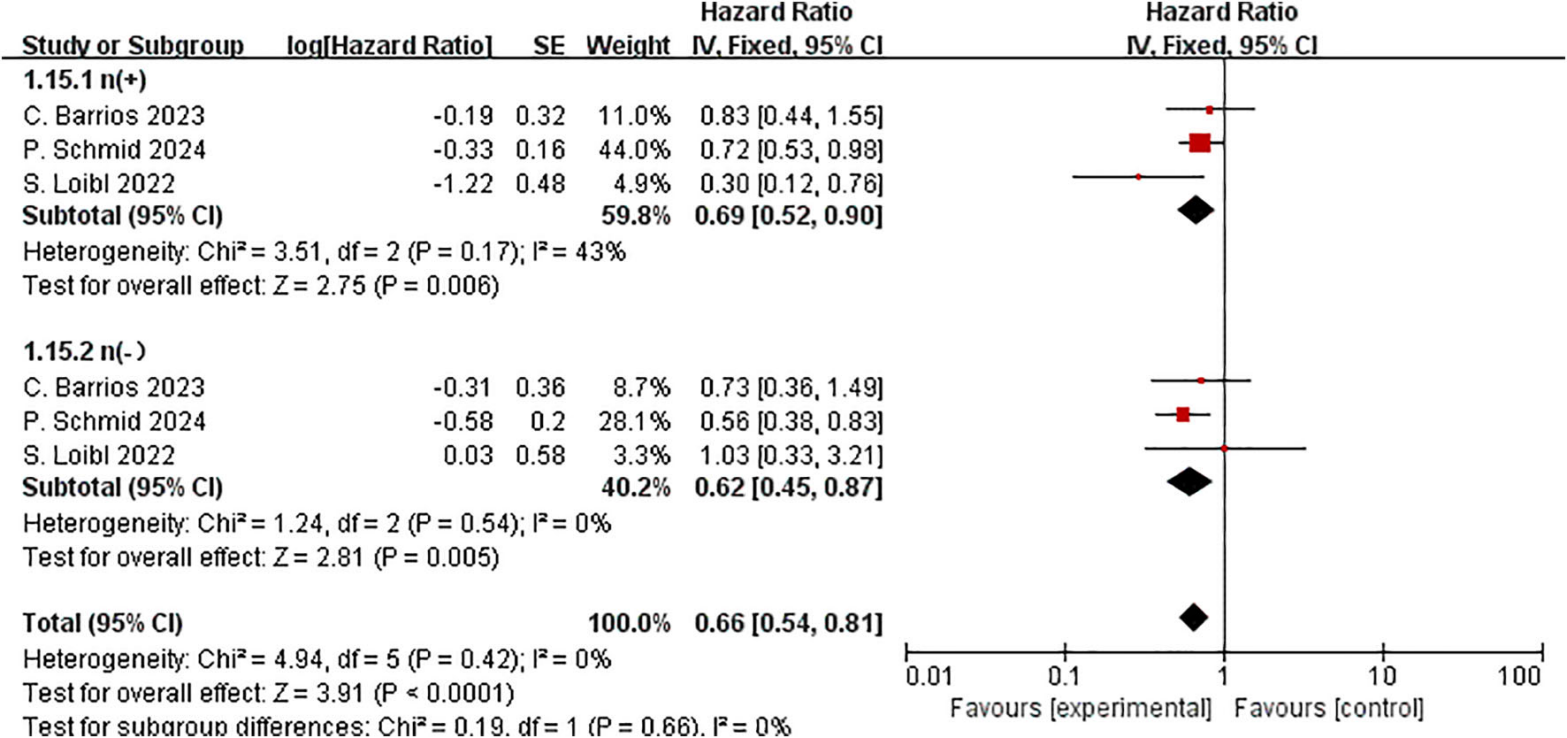
Forest plot of hazard ratios comparing EFS in patients for N stage subgroups.

On the basis of lymph node status, EFS was evaluated in three trials involving 1,679 participants. In the lymph node negative subgroup ([53.2%] 893 patients), the pooled HR was 0.62 (HR=0.62, 95% CI:0.45–0.87,P< 0.01,I^2^ = 0). In the lymph node positive subgroup ([46.8%]786 patients), the HR was 0.69 (HR=0.69, 95% CI:0.52–0.90,P < 0.01,I^2^ = 43%). There was no statistically significant difference in the two subgroups (P = 0.66) (**Figure 9**).

**Figure 10:**
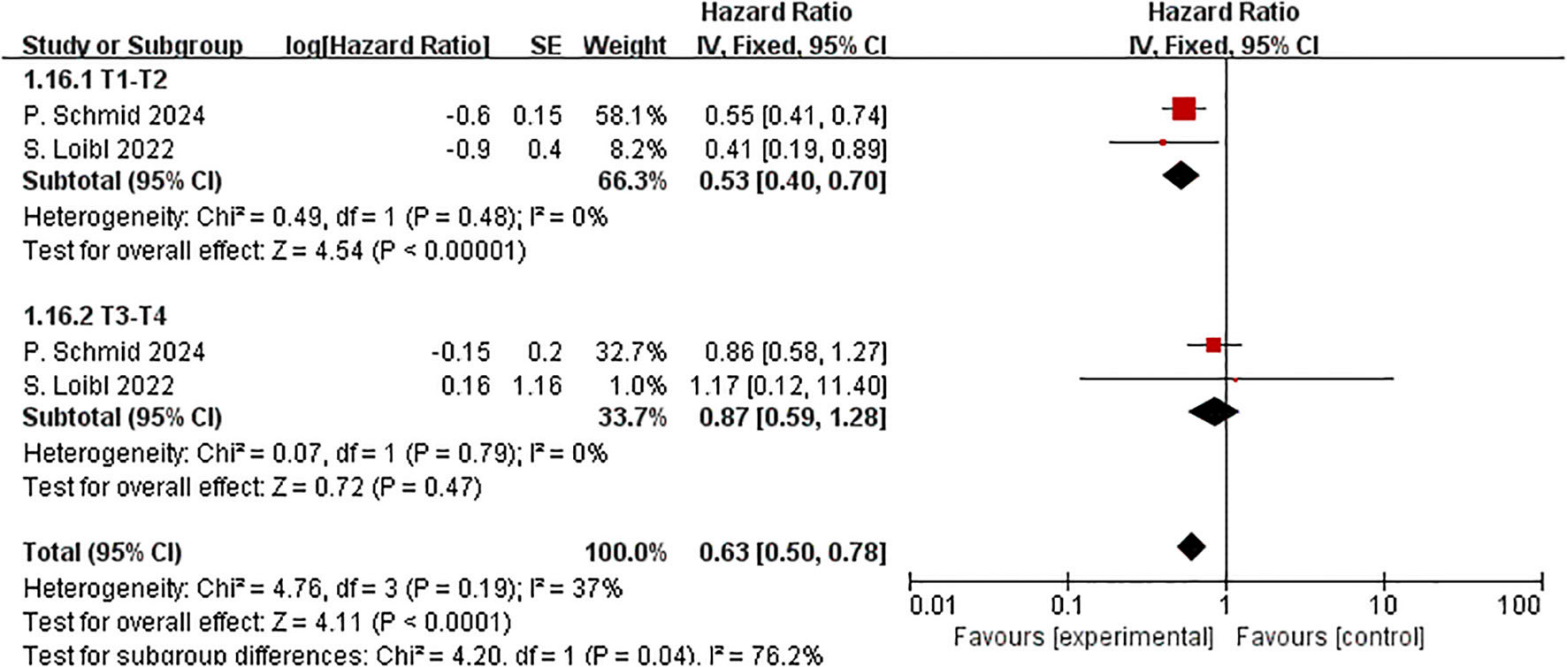
Forest plot of hazard ratios comparing EFS in patients for T stage subgroups.

T stage-based subgroup analysis for EFS was performed in two studies involving 1,348 individuals. The T1–2 stage comprised the majority of patients ([76.8%]1,035 patients). The analysis showed that patients with T1–2 stage breast cancer gained substantial EFS benefits due to the addition of ICIs (HR=0.53, 95% CI:0.40–0.70, P < 0.01, I^2^ = 0), while patients with T3-4stage cancer ([23.2%]313 patients) did not experience a significant benefit (HR=0.87, 95% CI:0.59–1.28, P =0.47, I^2^ = 0). Between the two subgroups, a nearly significant difference in EFS was noted. (P = 0.04) (**Figure 10**). The reduced event risk in early-stage tumor patients may indicate enhanced efficacy of immunotherapy when tumor burden is limited.

**Figure 11:**
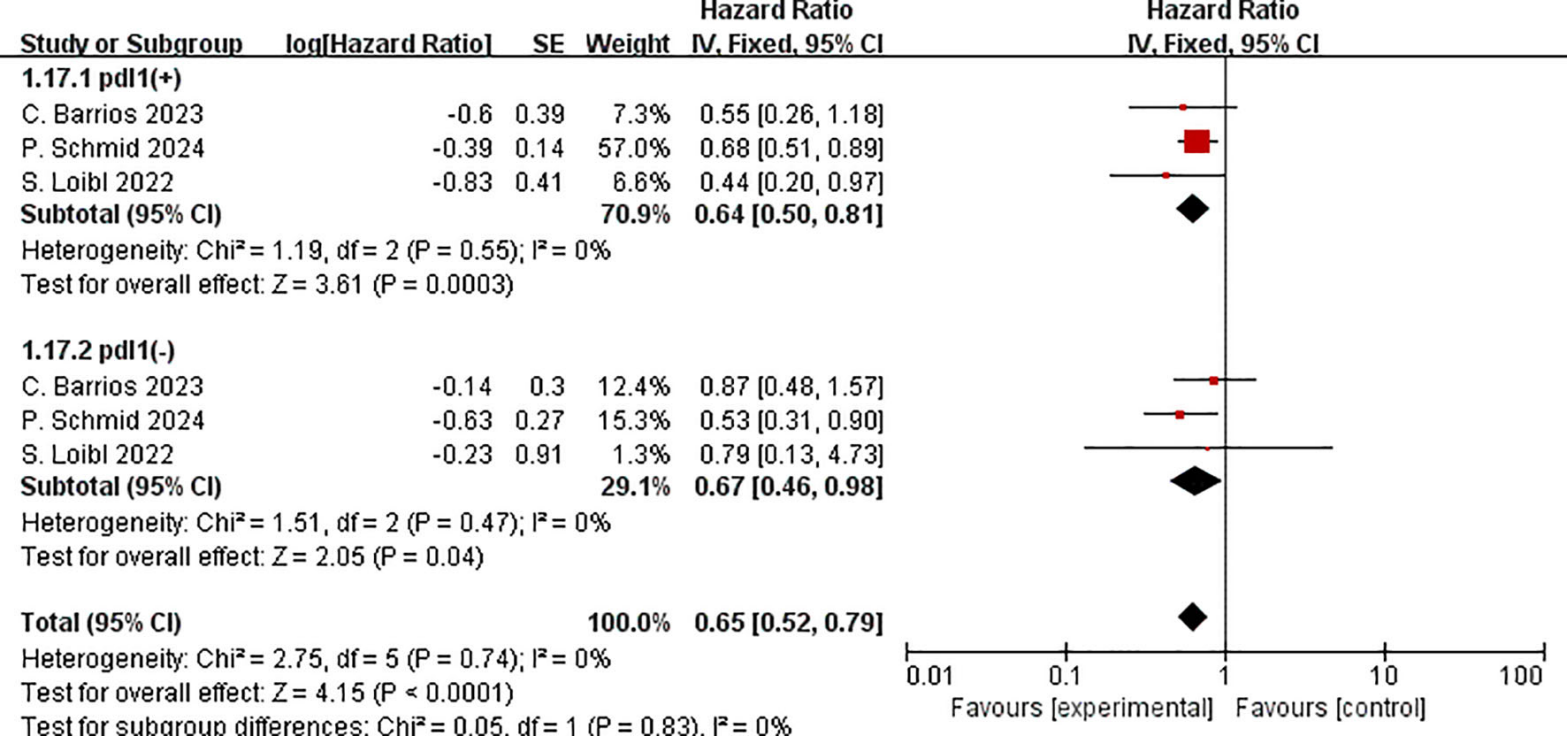
Forest plot of hazard ratios comparing EFS in patients for PD-L1 expression subgroups.

Immunocombination treatment increased EFS in both PD-L1 positive(HR=0.64,95% CI:0.50–0.81, P< 0.01, I^2^ = 0) and PD-L1 negative patients (HR=0.67, 95%CI:0.46–0.98, P =0.04, I^2^ = 0), according to a pooled analysis of three RCTs based on PD-L1 expression. No significant disparity was observed in the two groups. (P = 0.83) (**Figure 11**).

### 3.4 OS and subgroup analyses for PD-L1 positive patients

**Figure 12:**
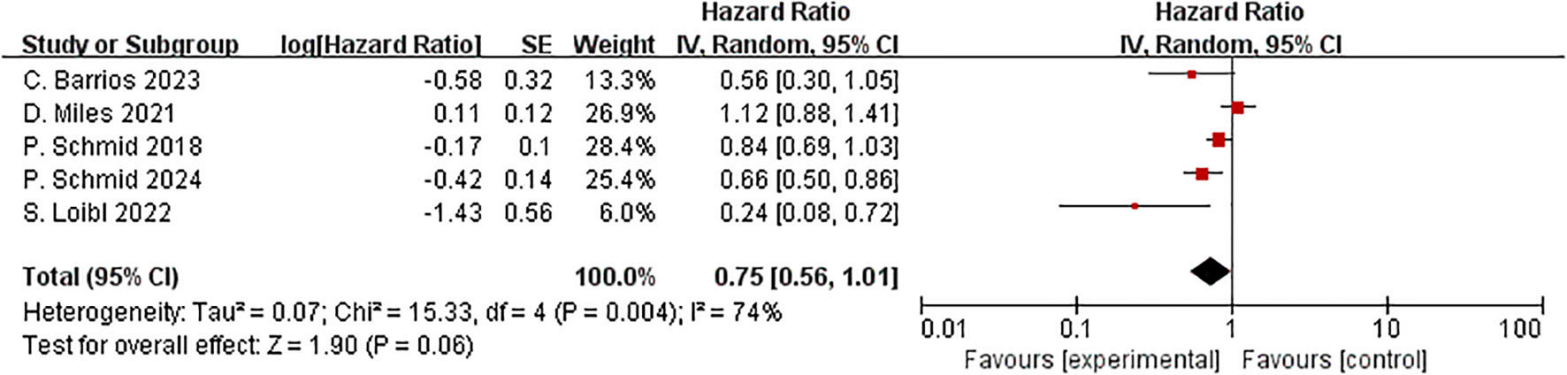
Forest plot of hazard ratios comparing OS in patients.

Five studies reported OS in the ITT and PD-L1 positive population subgroups. In the ITT population, ICIs plus neoadjuvant chemotherapy it is not significantly improved OS compared to neoadjuvant chemotherapy alone (HR=0.75, 95% CI:0.56–1.01, P =0.06, I2 = 74%) (**Figure 12**). For OS (I²=74%), heterogeneity likely stems from variations in post-progression therapies, differential PD-L1 assay thresholds, and study-specific follow-up durations, as explored in our subgroup analyses. This result indicates that ICIs have not improved the OS of ITT patients.

**Figure 13:**
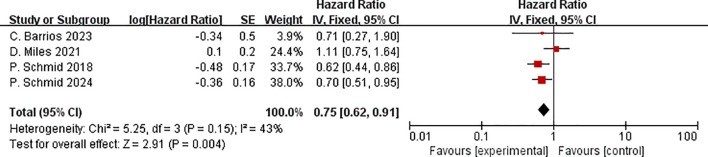
Forest plot of hazard ratios comparing OS in patients for PD-L1 expression subgroups.

The OS in the experimental group was statistically substantially superior to that in the control group in the PD-L1 positive population. (HR=0.75, 95% CI:0.62–0.91,P < 0.01, I^2^ = 43%) (**Figure 13**). While the treatment benefit was statistically significant in PD-L1+ patients, the overall ITT analysis showed a more modest effect with confidence intervals crossing unity.

### 3.5 PFS and subgroup analyses on PD-L1 positive

**Figure 14:**
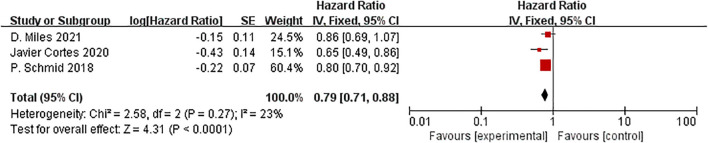
Forest plot of hazard ratios comparing PFS in patients.

Three studies reported PFS in the ITT and the PD-L1 positive population subgroups. In the ITT population, ICIs plus neoadjuvant chemotherapy significantly improved PFS compared to neoadjuvant chemotherapy alone (HR=0.79, 95% CI: 0.71-0.88, P < 0.01, I^2^ = 23%) **Figure 14**.

**Figure 15:**
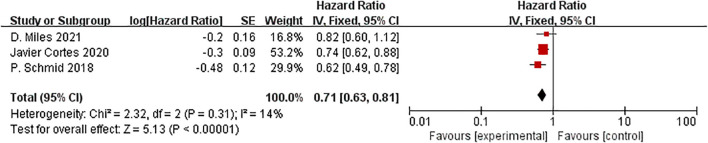
Forest plot of hazard ratios comparing PFS in patients for PD-L1 expression subgroups.

It had a statistically significant difference in the PFS between the experimental and control groups in the PD-L1 positive population.(HR=0.71, 95%CI:0.63-0.81, P<0.01, I^2^ = 14%) **Figure 15**.

### 3.6 Safety outcomes

**Figure 16:**
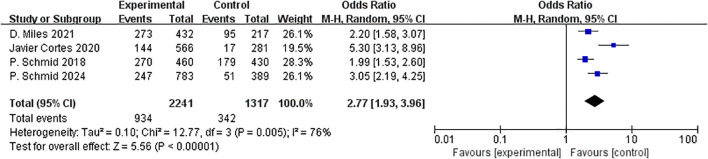
Forest plot of odds ratios for irAE.

Four studies included data on irAEs during treatment. Due to the high inter-study heterogeneity (I² = 76%), a random-effects model was employed for the analysis. The incidence of treatment-related irAEs was obviously higher in the ICIs group compared to the other group (OR=2.77, 95% CI:1.93–3.96, P < 0.01, I² = 76%) (**Figure 16**). Substantial heterogeneity observed for irAEs (I²=76%) may reflect differences in toxicity monitoring protocols across trials, variability in corticosteroid management, and distinct safety profiles of individual PD-1/PD-L1 inhibitors. Although the incidence of immune-related adverse events is higher, the survival benefits observed in PD-L1-positive subgroups may render this risk clinically acceptable for appropriately selected patients.

## 4 Discussion

The objective of this meta-analysis is to assess the efficacy and tolerability of ICIs, specifically PD-1/PD-L1 inhibitors when administered alongside neoadjuvant chemotherapy. The key endpoints under investigation encompass pCR, EFS, OS, PFS and irAEs. Our findings reveal that the integration of ICIs with neoadjuvant chemotherapy offers significant enhancements in pCR, EFS, OS, and PFS compared to neoadjuvant chemotherapy alone. However, this combination therapy also leads to a substantial rise in irAEs.

The integration of ICIs with neoadjuvant chemotherapy obviously elevates the rate in both pCR and EFS, with a 37% decrease in recurrence risk and a 77% rise in pCR occurrences in individuals with TNBC when compared to NACT alone, which is statistically significant. These RCTs show that adding ICIs to NACT significantly improves the effectiveness in the near term for patients with TNBC. Subgroup analysis of pCR reveals that the lymph node status is an important predictor of additional benefit from ICIs: while individuals without lymph node affection merely exhibit a non-significant trend of benefit, those with lymph node affection greatly benefit from additional ICIs. In lymph node positive patients, the administration of ICIs markedly boosts the rate of pCR. Patients with smaller primary tumors (T1-2) benefit better from ICIs in terms of EFS, according to subgroup analysis, with a huge 47% reduction in the rate of events. Conversely, patients with T3–4 stages do not obtain significant benefits. The T and N stages may impact the effectiveness of the treatment. For OS and PFS, compared to neoadjuvant chemotherapy alone, the PD-L1 positive population demonstrates notable advantages. Still, it is not significantly beneficial enough to the ITT population in OS, proving that the efficacy of ICIs has a close relationship with PD-L1 expression. The wide confidence intervals in the ITT analysis reflect the study’s limited power to detect smaller treatment effects in the overall population. Furthermore, Our results show that the occurrence of irAEs was significantly higher in the ICIs group than in the neoadjuvant chemotherapy group, showing that although ICIs increase therapeutic benefits, they significantly increase the risk of irAEs in patients. Research corroborates the same viewpoint, affirming the significant correlation between the efficacy of ICIs and irAEs in both the ITT and PD-L1 positive populations (25). However, what distinguishes our study is that we have incorporated more trials and introduced additional outcome indicators, such as pCR and EFS. This alerts us to the importance of assessing patient conditions, assessing the benefits and drawbacks and choosing suitable therapeutic approaches in clinical settings. These findings suggest future protocols should optimize patient stratification based on nodal status, T stage and PD-L1 expression while balancing efficacy and toxicity, advancing personalized TNBC treatment approaches.

Yuhan Wei (26) and Mittal (27) yielded similar results, suggesting that the addition of ICIs to neoadjuvant chemotherapy can improve the pCR and EFS in patients with TNBC. In contrast to these two studies, our analysis incorporated more recent clinical trial data with extended follow-up periods, providing updated efficacy evaluations. Furthermore, our safety analysis placed particular emphasis on irAEs, offering a more comprehensive assessment of the toxicity profile associated with this combination therapy.Mina (28) demonstrated the significant effect of ICIs on OS and PFS in both the PD-L1 positive and ITT populations, which is different from our study. Unlike this study, our research included more recent trial data with extended follow-up and performed additional lymph node subgroup analyses, offering deeper insights into treatment response variations among different patient subsets.

The existence of lymph node metastases is a major adverse prognostic factor in the context of breast cancer. Our meta-analysis’s findings, which include pCR and EFS from pertinent studies, further support the idea that patients with lymph node involvement can benefit greatly from immunotherapy as part of their treatment plan, making it a crucial therapeutic approach for this subgroup. Lymph nodes serve as critical “incubation hubs” for T-cell precursors during immunotherapy. The TCF-1+ SELL+ progenitor population differentiates into tumor-infiltrating exhausted T cells while maintaining long-term persistence. These LN-derived clones demonstrate multi-regional infiltration capacity, thereby sustaining durable anti-tumor immune responses during checkpoint blockade therapy (29). Among these, stem-like TCF-1+ CD8+ T cells in tumor-draining lymph nodes have emerged as pivotal players in ICI responses, rather than the exhausted T cell populations within the tumor microenvironment. According to research (30), the TCF-1+ CD8 T cell niche in tumors is extremely dynamic, moving between the tumor and surrounding lymphoid organs to facilitate intra-tumoral and systemic reactions. Their findings offer invaluable biological insights that complement our discoveries. By preventing the interaction between PD-1/PD-L1 in lymph nodes, PD-1/PD-L1 inhibitors rejuvenate the cytotoxic activity of T-cells, having a more notable salvage effect in this subgroup.

Simultaneously, the efficacy of PD-1/PD-L1 therapy varies according to the size of the primary tumor. Our meta-analysis, drawing insights from data on pCR and EFS, reveals that PD-1/PD-L1 demonstrates more pronounced efficacy in T1-T2 tumors compared to T3-T4 tumors. Researchers who studied melanoma patients concluded that a mismatch between T-cell renewal and tumor load is more likely to be the cause of ICIs’ clinical failure in numerous patients with larger malignancies than only their incapacity to stimulate immune renewal (31). Hypoxia can inhibit the sensitivity of cancer immunotherapy (32). The tumors often employ several strategies to evade host immune responses, including creating an immunosuppressive and hostile tumor environment, where hypoxia can lead to multimodal suppression of NK and NK-T cell responses. Larger primary tumor diameters tend to exacerbate hypoxia (33). Consequently, for patients with larger tumors (T3-4), combination therapy with ICIs might not be enough to improve the long term outcome; other successful treatment approaches, like combining therapy with targeting other immunosuppressive pathways or anti-angiogenic medications, may be required.

Meanwhile, in PD-L1 positive patients, ICIs treatment shows significant benefits in pCR, EFS, OS, and PFS, once again confirming the significant correlation between ICIs efficacy and PD-L1 expression. On the surface of tumors, PD-L1 engages with PD-1 on immune cells, primarily suppressing the effector functions of cytotoxic T lymphocytes, thereby facilitating Tumor growth and immune evasion (34). A high expression of PD-L1 is notably correlated with better clinical outcomes in patients receiving PD-L1 directed treatment (35). Consequently, ICIs therapy exhibits a more significant therapeutic effect in triple-negative breast cancer.

In ITT patients, PD-1/PD-L1 inhibitors often increase the incidence of irAEs, a phenomenon partly attributed to the potential cross-reactivity between tumor neoantigens and normal tissue antigens, which can trigger abnormal activation of the immune system (36). While this enhancement boosts the antitumor immune response, it may also compromise the tolerance of T cells towards normal self-tissue (37), ultimately leading to the occurrence of irAEs. Most irAEs are mild to moderate (Grade 1-2) and can be managed with corticosteroids or temporary treatment discontinuation. However, a small proportion of patients may develop severe (Grade 3-4) toxicities, requiring more potent immunosuppressive therapy and permanent discontinuation of the treatment. Early recognition and prompt intervention are therefore critical for effective irAEs management, as delayed treatment may lead to irreversible organ damage.

The short sample size and restricted data accessibility are the study’s primary drawbacks. As the aim was to compare the comparative effectiveness of different treatment options, only eight trials were included, and data availability was further restricted for certain subgroups. Additionally, specific information on the T and N stages for every patient was not available, and binary classification methods from the original publications were used for analysis, which may not represent the optimal predictive cutoff values. Furthermore, the ICIs used to treat patients with TNBC varied, leading PFS and OS studies to have slight and moderate heterogeneity. The limited quantity of included RCTs was insufficient to assess publication bias, and the combination of neoadjuvant chemotherapy regimens in the study and control groups was not completely consistent across studies, with variations in PD-L1 detection methods and ICI agents, also present. These factors may all introduce potential bias into the results and contribute to significant heterogeneity in some outcomes. While our meta-analysis provides insights into the efficacy and safety of PD-1/PD-L1 inhibitors in TNBC, several important questions remain unanswered. The heterogeneity in PD-L1 testing methods across studies underscores the need for standardized assays to improve patient selection and outcome interpretation. Additionally, further research is needed to identify reliable predictors of immune-related adverse events and to explore the potential of combining immunotherapy with novel biomarkers beyond PD-L1, such as tumor-infiltrating lymphocytes or genomic signatures, particularly in patients who show limited response to current regimens. Despite these limitations, this meta-analysis offers the most recent exploratory study on the clinical traits linked to TNBC and pCR, EFS, PFS, OS, and irAEs. It also serves as a crucial foundation for the design of clinical judgment and future clinical trials.

## 5 Conclusion

The present analysis evaluates the effectiveness and tolerability of combining PD-1/PD-L1 inhibitors with neoadjuvant chemotherapy for the treatment of TNBC. Key findings show that, as opposed to just neoadjuvant chemotherapy, the combination significantly enhances pCR, EFS, OS(PD-L1 positive population), and PFS, but also increases irAEs. Lymph node status influences immunotherapy benefits, with limited additional benefits seen in node-negative patients. While T stage trends suggest greater EFS benefit at lower stages, it is not yet a recommended predictive biomarker. Overall, these results guide future trial design, economic analysis, and clinical practice in TNBC treatment.

## Data Availability

The raw data supporting the conclusions of this article will be made available by the authors, without undue reservation.
